# Venous thromboembolism prophylaxis after anterior cruciate ligament reconstruction: retrospective case-control study

**DOI:** 10.1051/sicotj/2025032

**Published:** 2025-07-16

**Authors:** Hatem B. Afana, Shine Ashokan, Thomas Nau

**Affiliations:** 1 Orthopaedic Department, King’s College Hospital London Dubai Dubai Hills, Alkhail Road, Marabea’ East Exit PO Box No 340901 Dubai United Arab Emirates; 2 Mohammed Bin Rashid University of Medicine and Health Sciences Building 14, Dubai Healthcare City PO Box 505055 Dubai United Arab Emirates

**Keywords:** ACL reconstruction, Thromboprophylaxis, Deep vein thrombosis, Venous thromboembolism

## Abstract

*Introduction*: Venous thromboembolism (VTE) is a rare but potentially serious complication following anterior cruciate ligament reconstruction (ACLR). There is no guideline for the routine use of anticoagulants post-ACLR surgery. *Methods*: This retrospective case-control study reviewed 199 patients who underwent ACLR between February 2020 and November 2024. Two groups were compared: Group A (*n* = 113) received no pharmacological prophylaxis, while Group B (*n* = 86) received low-molecular-weight heparin (LMWH) for 2 weeks postoperatively. The incidence of symptomatic VTE, postoperative bleeding, and related complications was evaluated. *Results*: No symptomatic VTE or bleeding complications were observed in either group. There was no statistically significant difference between the groups in terms of age, BMI, smoking, comorbidities, and postoperative weight bearing. There was a significant difference in surgical duration, graft type, and meniscal procedure. *Discussion:* Our findings support a risk-stratified approach rather than universal pharmacologic prophylaxis in ACLR patients.

## Introduction

Anterior cruciate ligament (ACL) injuries are among the most frequent orthopaedic injuries encountered in young, active individuals [1]. Anterior cruciate ligament reconstruction (ACLR) has become the standard surgical treatment for ACL tear, particularly in younger and/or more active patients, for restoring knee stability and function, with consistently high rates of return to sports and favourable long-term outcomes, especially with early reconstruction. [2]. Despite being commonly performed as a same-day procedure with low morbidity, ACLR involves multiple risk factors that may contribute to venous thromboembolism (VTE), including the use of a tourniquet, limited postoperative mobility, and surgical duration.

Venous thromboembolism, comprising deep vein thrombosis (DVT) and pulmonary embolism (PE), is a potentially life-threatening complication. However, the true incidence of VTE after ACLR is not well defined. Reported rates of symptomatic VTE following ACLR range from 0.53% to 1.22%, with large registry-based studies and meta-analyses suggesting an overall low risk [3, 4]. Nonetheless, the incidence of asymptomatic DVT may be significantly higher, reported in up to 6.6% to 10.8% of patients in some retrospective studies using routine Doppler ultrasound screening [5–8].

This discrepancy raises an important clinical dilemma: should asymptomatic thrombotic events drive the use of routine pharmacologic thromboprophylaxis in ACLR patients? Current clinical practice is highly variable. While major orthopaedic procedures such as hip and knee arthroplasty have established protocols for anticoagulation, ACLR is not universally recognized as a high-risk procedure. Guidelines from the American College of Chest Physicians (ACCP) [9] do not recommend routine thromboprophylaxis following arthroscopic procedures. However, no specific recommendations exist for ACLR, and the decision to prescribe anticoagulation is often left to individual clinical judgment.

Moreover, thromboprophylaxis carries risks of its own. Use of low-molecular-weight heparin (LMWH) or other anticoagulants may lead to postoperative bleeding, hematoma formation, and joint stiffness [10, 11]. These complications are particularly undesirable in the context of ACLR, where early rehabilitation and restoration of range of motion are critical for optimal outcomes. Previous studies have shown conflicting results on whether pharmacologic prophylaxis meaningfully reduces symptomatic VTE without increasing adverse events. [12–15].

In previous literature, the main risk factors for VTE after ACL reconstruction include: age >35 years, tobacco use, high BMI, non-weight bearing and prolonged tourniquet time [3, 7, 8, 14, 16, 17].

Considering this uncertainty, there is a need for evidence-based guidance on the role of thromboprophylaxis following ACL reconstruction. Our study aims to assess the real-world incidence of symptomatic VTE and postoperative bleeding complications in a cohort of ACLR patients, comparing outcomes between those who received routine LMWH and those who did not receive any chemical prophylaxis. We also explore the influence of patient-specific surgical factors, including graft choice, meniscal procedures, tourniquet use and postoperative weight bearing, on postoperative complications.

We hypothesized that symptomatic VTE events would be rare in both groups without a significant difference between those patients who received routine LMWH and those who did not.

## Materials and methods

### Level III (retrospective case-control study)

This retrospective cohort study included all patients who underwent primary ACLR at a single tertiary orthopaedic centre between February 2020 and November 2024. Institutional review board approval was obtained prior to data collection.

Patients were identified through the hospital’s electronic surgical records. Inclusion criteria were: (1) age ≥16 years, (2) primary ACLR performed by one of two fellowship-trained sports medicine surgeons, and (3) complete follow-up of at least 12 weeks. Exclusion criteria included multiligament knee injuries, revision ACLR, pre-existing VTE, anticoagulation therapy prior to surgery, and incomplete records.

Patients were divided into two groups based on the routine postoperative practice of the operating surgeon. This non-randomized design introduces a potential for selection bias:

- Group A (*n* = 113): ACLR performed with hamstring tendon autografts, no routine chemical thromboprophylaxis prescribed.
- Group B (*n* = 86): ACLR performed with quadriceps tendon autografts, with standard administration of LMWH for 14 days postoperatively.

All patients received mechanical compression stockings and were encouraged to begin early weight-bearing and mobilization on the first postoperative day. No patients in either group received aspirin or direct oral anticoagulants. The surgical technique in both groups included arthroscopic ACLR with femoral and tibial tunnel placement using standardized instrumentation. Meniscal procedures were performed as indicated, including partial meniscectomy or repair.

The following data were collected from medical records and operative notes:

- Demographics: age, gender, body mass index (BMI), smoking status, and comorbidities (e.g., diabetes, cardiovascular disease).
- Surgical details: graft type (hamstring, quadriceps tendon), tourniquet use and duration, meniscus procedure type (meniscectomy, repair, none).
- Postoperative management: use of chemical thromboprophylaxis, duration, use of compression stockings, and early mobilization protocol.
- Outcomes: symptomatic VTE (confirmed DVT or PE), postoperative bleeding requiring intervention, reoperation for any cause, and joint effusion requiring aspiration.

VTE events were defined as symptomatic DVT or PE confirmed by duplex ultrasonography or CT pulmonary angiography during the follow-up period. Bleeding was defined as any clinically evident postoperative haemorrhage or hematoma requiring aspiration, wound revision, or delay in physiotherapy. Reoperations were defined as any unplanned surgical procedures within 12 weeks of the index ACLR.

Statistical analysis was conducted using Python (SciPy and pandas libraries). Continuous variables (e.g., age, BMI, surgical time) were compared using independent *t*-tests, and categorical variables (e.g., gender, smoking, meniscus procedures, VTE events) were analyzed using chi-square tests. A *p*-value < 0.05 was considered statistically significant. A 95% confidence interval (CI) is considered statistically significant when it does not include the null value.

## Results (Table 1)

A total of 199 patients who underwent primary ACL reconstruction were included in the final analysis. Of these, 113 patients (57%) were in Group A (no chemical prophylaxis) and 86 patients (43%) were in Group B (received LMWH for 14 days). All patients were followed for a minimum of 12 weeks postoperatively.

**Table 1:** Descriptive summary of patient data collected from February 2020 to November 2024.

	Group A	Group B	Total
Number of cases			
Total cases	125 patients	91 pa	216 patients
Excluded	12 patients	5 patients	17 patients
Included	113 patients	86 patients	199 patients
Average age	33.3 years	35 years	34.15 years
Average BMI	27.36 kg/m^2^	26.75 kg/m^2^	27 kg/m^2^
Gender			
Male	92 patients	59 patients	151 patients
Female	21 patients	27 patients	48 patients
Smokers	8 patients	4 patients	12 patients
Chronic diseases	12 patients	13 patients	25 patients
Mean surgical duration	93.4 minutes	75.3 minutes	84.35 min
Autograft*			
Hamstrings tendon	98 cases	0 cases	98 cases
BTB	13 cases	0 cases	13 cases
PTQT	2 cases	86 cases	88 cases
Meniscal surgery			
Meniscal repair	40 cases	12 cases	52 cases
Partial meniscectomy	32 cases	37 cases	69 cases
No meniscal surgery	41 cases	37 cases	78 cases
Postoperative weight bearing			
FWB	91 cases	77 cases	168 cases
PWB	20 cases	8 cases	28 cases
Non-weight bearing	2 cases	1 case	3 cases

BMI = body mass index, BTB = bone-patellar tendon-bone, PTQT = partial-thickness quadriceps tendon, FWB = full weight bearing, PWB = partial weight bearing.

* Graft type is potential confounded with prophylaxis use (hamstring, BTB vs. quadriceps).

### Demographics

There was no statistically significant difference between groups in terms of age (Group A: 33.2 ± 9.2 years; Group B: 34.8 ± 9.7 years; *p* = 0.233) or BMI (Group A: 27.3 ± 4.3 kg/m^2^; Group B: 26.7 ± 4.4 kg/m^2^; *p* = 0.355). Gender distribution showed a trend towards significant difference, with males predominating in both groups (Group A: 92 males/21 females; Group B: 59 males/27 females; *p* = 0.054). No statistically significant difference was observed in smoking status. (Group A: 11 smokers; Group B: 4 smokers; *p* = 0.324), or in documented comorbidities (Group A: 12 patients; Group B: 13 patients; *p* = 0.343055)

### Surgical characteristics

Tourniquet use was standard in all cases. The mean surgical time was significantly longer in Group A (93.5 ± 22.9 min) compared to Group B (75.3 ± 15.4 min; *p* < 0.001).

Graft types differed significantly between groups: Group A patients predominantly received hamstring tendon autografts, while Group B patients received quadriceps tendon autografts exclusively (*p* < 0.001). Graft type was not randomized and was fully confounded with prophylaxis strategy (i.e., hamstring grafts did not receive prophylaxis, quadriceps grafts received prophylaxis).

Meniscal procedures were also significantly different between groups:

- Meniscus repairs: Group A = 40, Group B = 12 (*p* < 0.001).
- Meniscectomies: Group A = 31, Group B = 37 (*p* = 0.032).
- No meniscal procedure: Group A = 42, Group B = 37 (*p* = 0.490).

### Postoperative weight bearing

- Full weight bearing (FWB): Group A = 91, Group B= 77 (*p* = 0.082733).
- Partial weight bearing (PWB): Group A =20, Group B = 8 (*p* = 0.091502).
- Non weight bearing (NWB): Group A =2, Group B = 1 (*p* = 0.727706).

## Clinical outcomes

No cases of symptomatic VTE were reported in either group during the follow-up period. The 95% confidence interval for the difference in DVT rates between the prophylaxis and no-prophylaxis groups is approximately −2.65% to 3.49%.

There were also no instances of postoperative bleeding or joint effusion requiring aspiration in group B, in which LMWH was administered. Notably, one patient in group A, who did not receive LMWH, developed an effusion which required arthroscopic washout.

Overall, the incidence of postoperative complications was low across both groups, with no evidence that routine use of chemical thromboprophylaxis reduced the risk of symptomatic VTE.

## Discussion

This retrospective study directly compared patients undergoing ACLR with and without chemical thromboprophylaxis, offering a focused evaluation of postoperative VTE risk. While previous studies have compared patient groups with and without pharmacologic thromboprophylaxis, our study is among the first to do so using two clearly defined cohorts managed under routine clinical conditions, with standardized administration of LMWH for 14 days in one group and no prophylaxis in the other. Despite differences in surgical duration, graft type and meniscal management between the groups, our results demonstrated no symptomatic VTE events in either cohort.

These findings support earlier work showing that symptomatic VTE is exceedingly rare after ACLR. This finding may be attributed to the routine use of early mobilization postoperatively and routine muscle contraction exercises of the foot and calf muscles, which are effective in reducing the risk of DVT and were used in all patients in this study [18, 19].

McIntire et al. reported no symptomatic VTE in 1233 adolescent ACLR patients in those who received full-dose aspirin chemoprophylaxis versus those with no chemoprophylaxis, [14] and Gaskill et al. observed a symptomatic VTE rate of just 0.53% in a large cohort that included 16,558 cases post-ACLR [4]. Although Joo et al. reported an 8.1% rate of asymptomatic DVT in ACLR patients screened postoperatively, none of those patients developed symptomatic PE, suggesting that asymptomatic thrombi may not carry the same clinical relevance [6].

The absence of VTE in both groups of our study underscores that routine anticoagulation may not be necessary for all patients undergoing ACLR. Importantly, our results showed that no cases of VTE, bleeding, joint effusion, or reoperation were observed in either group. While this suggests a low overall complication rate, the absence of events limits our ability to draw definitive conclusions about the impact of chemical prophylaxis on these outcomes. While one patient in the non-anticoagulated group required reoperation, this was unrelated to thrombotic or hemorrhagic events and did not reach statistical significance.

In our cohort, a statistically significant difference was observed in the distribution of meniscal procedures between groups. While this finding is noteworthy, it should be interpreted cautiously, as the study was not powered to examine the independent effects of meniscal treatment type. Future research could explore whether meniscal procedures independently affect thromboembolic outcomes.

Our study also highlights the potential influence of surgical factors on thromboprophylaxis decisions. Although prior studies have suggested that avoiding tourniquet use may reduce the incidence of postoperative DVT and pain [20, 21], a tourniquet was used for all cases in both groups. Moreover, Group A included more cases with meniscus repairs, resulting in longer operative times, and more post-operative partial weight bearing. Despite this higher surgical burden, the group without pharmacologic prophylaxis did not demonstrate any increased thrombotic risk. These findings raise questions about whether chemical thromboprophylaxis should be reserved for high-risk patients aged >35 years, tobacco use, high BMI, non-weight bearing and prolonged tourniquet time [3, 7, 8, 14, 16, 17], rather than universally applied.

Prior meta-analyses have shown mixed results. Li et al. and Zhu et al. found that LMWH reduces the incidence of symptomatic and asymptomatic DVT in knee arthroscopy, yet their findings also note a concurrent increase in bleeding-related complications [13, 22]. Gu et al. and Qin et al. reported that prophylaxis may contribute to postoperative stiffness, hemarthrosis, and delays in rehabilitation [10, 11]. In ACLR patients where early recovery of range of motion is critical, these risks may outweigh the modest theoretical benefit of anticoagulation.

The current study has several limitations. First, as a retrospective design. Second, selection bias may exist, particularly since treatment groups were defined by surgeon preference. This approach limits the internal validity and comparability between groups. However, this real-world structure also strengthens the relevance of our findings to everyday practice. Third, we did not screen for asymptomatic DVTs with ultrasound, meaning some subclinical thrombi may have been missed. However, we focused deliberately on symptomatic, clinically meaningful outcomes. Fourth, an important limitation is the complete confounding between graft type and prophylaxis regimen.

Despite these limitations, the strength of our study lies in its head-to-head comparison of two common postoperative pathways in a real-world clinical setting. Based on our findings, we support a selective, risk-adapted approach to chemical thromboprophylaxis in ACLR. Routine anticoagulation may not be warranted in low-risk patients and could potentially expose them to unnecessary harm.

Future directions could include identifying validated clinical scores or genetic risk scores to better stratify patients who may benefit from chemical prophylaxis. With ongoing advancements, genetic testing is expected to become more accessible and routinely integrated into clinical care. This may allow for early identification of individuals with inherited thrombophilic tendencies, offering a personalized approach to thromboprophylaxis in ACL reconstruction. Until such tools are available, mechanical prophylaxis and early mobilization remain cornerstones of VTE prevention following ACL reconstruction.

## Conclusion

In this retrospective analysis of 199 patients who underwent ACLR, no cases of symptomatic VTE, bleeding, joint effusion, or reoperation were observed in either the prophylaxis or non-prophylaxis groups. These results indicate that routine administration of chemical thromboprophylaxis may not be necessary for low-risk individuals undergoing ACLR.

Further prospective studies and risk-stratification tools are needed to define which patients may truly benefit from chemical thromboprophylaxis after ACL surgery.

## Data Availability

The authors state that they have full control of all primary data and that they agree to allow the journal to review their data if requested.
